# 4-(Oct­yloxy)phenyl 2-oxo-2*H*-chromene-3-carboxyl­ate

**DOI:** 10.1107/S1600536813000214

**Published:** 2013-01-09

**Authors:** B. S. Palakshamurthy, S. Sreenivasa, H. T. Srinivasa, K. R. Roopashree, H. C. Devarajegowda

**Affiliations:** a Department of Physics, Yuvaraja’s College (Constituent College), University of Mysore, Mysore 570 005, Karnataka, India; b Department of Studies and Research in Chemistry, Tumkur University, Tumkur 572 103, Karnataka, India; c Raman Research Institute, C. V. Raman Avenue, Sadashivanagar, Bangalore 560 080, Karnataka, India

## Abstract

In the title compound, C_24_H_26_O_5_, the 2*H*-chromene ring system is essentially planar, with a maximum deviation of 0.029 (2) Å from the best-fit mean plane incorporating both rings. The dihedral angle between the 2*H*-chromene ring system and the benzene ring is 21.00 (1)°. In the crystal, pairs of C—H⋯O hydrogen bonds generate an *R*_2_^2^(8) ring pattern. These contacts are bolstered by weaker bifurcated C—H⋯O hydrogen bonds.

## Related literature

For general background to coumarin derivatives and their biological and technological applications, see: Georgieva *et al.* (2004 ▶); Creaven *et al.* (2005 ▶); Morita *et al.* (2005 ▶); Tian *et al.* (2003 ▶); Iliopoulos *et al.* (2010 ▶); Hejchman *et al.* (2011 ▶). For hydrogen-bond motifs, see: Bernstein *et al.* (1995 ▶).
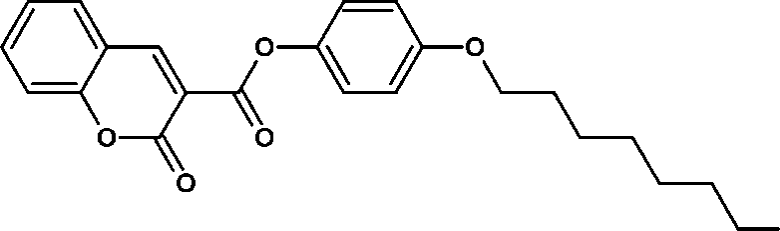


## Experimental

### 

#### Crystal data

C_24_H_26_O_5_
*M* *_r_* = 394.45
Monoclinic, 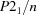

*a* = 14.464 (3) Å
*b* = 6.7548 (15) Å
*c* = 21.381 (5) Å
β = 91.663 (8)°
*V* = 2088.0 (8) Å^3^
*Z* = 4
Mo *K*α radiation
μ = 0.09 mm^−1^
*T* = 293 K
0.29 × 0.25 × 0.21 mm

#### Data collection

Bruker SMART CCD area-detector diffractometer
Absorption correction: multi-scan (*SADABS*; Sheldrick, 2007 ▶) *T*_min_ = 0.975, *T*_max_ = 0.982
22609 measured reflections
3615 independent reflections
1926 reflections with *I* > 2σ(*I*)
*R*_int_ = 0.084

#### Refinement

*R*[*F* ^2^ > 2σ(*F* ^2^)] = 0.058
*wR*(*F* ^2^) = 0.146
*S* = 0.94
3615 reflections
264 parameters
H-atom parameters constrained
Δρ_max_ = 0.18 e Å^−3^
Δρ_min_ = −0.14 e Å^−3^

### 

Data collection: *SMART* (Bruker, 2001 ▶); cell refinement: *SAINT* (Bruker, 2001 ▶); data reduction: *SAINT*; program(s) used to solve structure: *SHELXS97* (Sheldrick, 2008 ▶); program(s) used to refine structure: *SHELXL97* (Sheldrick, 2008 ▶); molecular graphics: *ORTEP-3* (Farrugia, 2012 ▶); software used to prepare material for publication: *SHELXL97*.

## Supplementary Material

Crystal structure: contains datablock(s) I, global. DOI: 10.1107/S1600536813000214/sj5291sup1.cif

Structure factors: contains datablock(s) I. DOI: 10.1107/S1600536813000214/sj5291Isup2.hkl

Supplementary material file. DOI: 10.1107/S1600536813000214/sj5291Isup3.cml

Additional supplementary materials:  crystallographic information; 3D view; checkCIF report

## Figures and Tables

**Table 1:** Hydrogen-bond geometry (Å, °)

*D*—H⋯*A*	*D*—H	H⋯*A*	*D*⋯*A*	*D*—H⋯*A*
C4—H4⋯O1^i^	0.93	2.59	3.513 (4)	174
C9—H9⋯O2^i^	0.93	2.51	3.420 (3)	167
C16—H16⋯O2^i^	0.93	2.71	3.551 (3)	151
C16—H16⋯O3^i^	0.93	2.63	3.338 (3)	133

Symmetry code: (i) 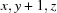.

**Fig. 1.**
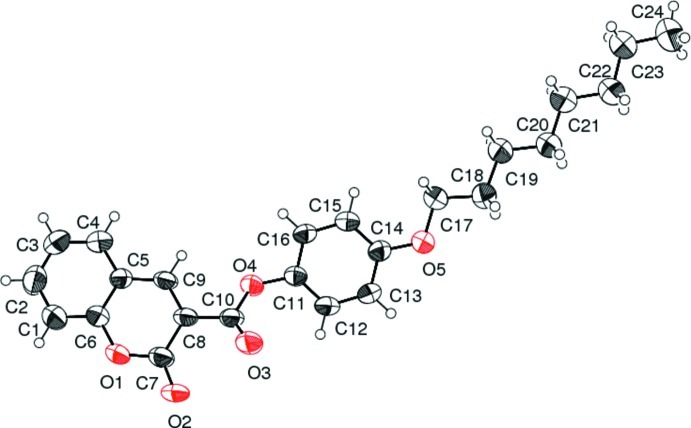
The molecular structure of the title compound. Displacement ellipsoids are drawn at the 50% probability level. Hydrogen atoms are shown as spheres of arbitrary radius.

**Fig. 2.**
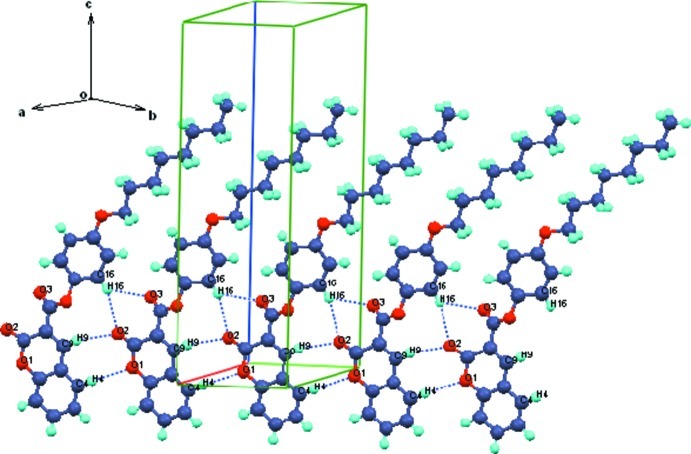
Crystal packing for the title compound with hydrogen bonds drawn as dashed lines.

## supplementary crystallographic information

### Comment

Coumarin derivatives have been attracted increasing attention due to their extensive biological applications, such as anticancer, anti-inflammatory and anticoagulant agents (Georgieva *et al.*, 2004; Creaven *et al.*, 2005). The coumarin nucleus has been the focus of our recent research concerning the design, synthesis and characterization to investigate their liquid crystal properties together with crystal structure studies (Morita *et al.*, 2005; Tian *et al.*, 2003). Coumarins are interesting class of heterocycles because of their dipolar moment increases by external stimulus such as light, temperature, electric current and chemical reaction (Iliopoulos *et al.*, 2010). The excitation of the coumarin chromophore increases the electron density of its carbonyl groups owing to excited photochemical and photophysical properties such as molecular fluorescent sensors, laser dyes and many industrial applications (Hejchman *et al.*, 2011).

The asymmetric unit of 4-(octyloxy)phenyl 2-oxo-2*H*-chromene-3 -carboxylate is shown in Fig. 1. The 2*H*-chromene ring (O1/C1–C9) system is planar, with a maximum deviation of 0.028 (2) Å for atom C8. The dihedral angle between 2*H*-chromene ring (O1/C1–C9) and benzene ring (C11–C16) is 21.11 (1)°. The crystal structure is characterized by intermolecular C4—H4···O1 and C9—H9···O2 hydrogen bonding generating an *R*_2_^2^(8) ring pattern (Bernstein *et al.*, 1995). Bifurcated C16—H16···O2 and C16—H16···O3 contacts further strengthen the packing, Fig. 2.

### Experimental

A mixture of 2-oxo-2*H*-chromene-3-carboxylic acid (19 mg,1 mmol), 4-(octyloxy)phenol (22.2 mg,1 mmol), N,*N* -dicyclohexylcarbodiimide (23 mg,1.2 mmol) and a catalytic quantity of *N*,*N*-dimethylaminopyrimidine was stirred in 5 ml of dry dichloromethane for 24 h at room temperature. The residue obtained on removal of solvent was chromatographed on silica gel and eluted with chloroform. Removal of solvent from the eluate afforded a colorless solid, which was re-crystallized from absolute ethanol to obtain needle like crystals of the title compound for X-ray diffraction analysis.

### Refinement

All H atoms were positioned geometrically, with C—H = 0.93 Å for aromatic H, C—H = 0.97 Å for methylene H and C—H = 0.96 Å for methyl H, and refined using a riding model with *U*_iso_(H) = 1.5*U*_eq_(C) for methyl H and *U*_iso_(H) = 1.2*U*_eq_(C) for all other H.

### Crystal data

**Table:**

C_24_H_26_O_5_	*F*(000) = 840
*M**_r_* = 394.45	*D*_x_ = 1.255 Mg m^−^^3^
Monoclinic, *P*2_1_/*n*	Melting point: 580 K
Hall symbol: -P 2yn	Mo *K*α radiation, λ = 0.71073 Å
*a* = 14.464 (3) Å	Cell parameters from 3615 reflections
*b* = 6.7548 (15) Å	θ = 3.2–25.0°
*c* = 21.381 (5) Å	µ = 0.09 mm^−^^1^
β = 91.663 (8)°	*T* = 293 K
*V* = 2088.0 (8) Å^3^	Needles, colourless
*Z* = 4	0.29 × 0.25 × 0.21 mm

### Data collection

**Table:**

Bruker SMART CCD area-detector diffractometer	3615 independent reflections
Radiation source: fine-focus sealed tube	1926 reflections with *I* > 2σ(*I*)
Graphite monochromator	*R*_int_ = 0.084
ω and φ scans	θ_max_ = 25.0°, θ_min_ = 3.2°
Absorption correction: multi-scan (*SADABS*; Sheldrick, 2007)	*h* = −17→16
*T*_min_ = 0.975, *T*_max_ = 0.982	*k* = −8→5
22609 measured reflections	*l* = −25→25

### Refinement

**Table:**

Refinement on *F*^2^	Secondary atom site location: difference Fourier map
Least-squares matrix: full	Hydrogen site location: inferred from neighbouring sites
*R*[*F*^2^ > 2σ(*F*^2^)] = 0.058	H-atom parameters constrained
*wR*(*F*^2^) = 0.146	*w* = 1/[σ^2^(*F*_o_^2^) + (0.077*P*)^2^] where *P* = (*F*_o_^2^ + 2*F*_c_^2^)/3
*S* = 0.94	(Δ/σ)_max_ < 0.001
3615 reflections	Δρ_max_ = 0.18 e Å^−^^3^
264 parameters	Δρ_min_ = −0.14 e Å^−^^3^
0 restraints	Extinction correction: *SHELXL97* (Sheldrick, 2008), Fc^*^=kFc[1+0.001xFc^2^λ^3^/sin(2θ)]^-1/4^
Primary atom site location: structure-invariant direct methods	Extinction coefficient: 0.0019 (7)

### Special details

**Table:**

Geometry. All s.u.'s (except the s.u. in the dihedral angle between two l.s. planes) are estimated using the full covariance matrix. The cell s.u.'s are taken into account individually in the estimation of s.u.'s in distances, angles and torsion angles; correlations between s.u.'s in cell parameters are only used when they are defined by crystal symmetry. An approximate (isotropic) treatment of cell s.u.'s is used for estimating s.u.'s involving l.s. planes.
Refinement. Refinement of *F*^2^ against ALL reflections. The weighted *R*-factor *wR* and goodness of fit *S* are based on *F*^2^, conventional *R*-factors *R* are based on *F*, with *F* set to zero for negative *F*^2^. The threshold expression of *F*^2^ > 2σ(*F*^2^) is used only for calculating *R*-factors(gt) *etc*. and is not relevant to the choice of reflections for refinement. *R*-factors based on *F*^2^ are statistically about twice as large as those based on *F*, and *R*- factors based on ALL data will be even larger.

### Fractional atomic coordinates and isotropic or equivalent isotropic displacement parameters (Å^2^)

**Table:**

	*x*	*y*	*z*	*U*_iso_*/*U*_eq_	
O1	0.11082 (14)	0.0294 (3)	−0.01675 (8)	0.0705 (6)	
O2	0.11756 (15)	−0.1104 (3)	0.07552 (9)	0.0826 (7)	
O3	0.06844 (14)	0.1538 (3)	0.17685 (9)	0.0744 (6)	
O4	0.14523 (12)	0.4373 (2)	0.16091 (7)	0.0610 (5)	
O5	0.14206 (14)	0.7392 (3)	0.40100 (8)	0.0730 (6)	
C1	0.1075 (2)	0.1595 (5)	−0.11861 (13)	0.0755 (9)	
H1	0.1043	0.0314	−0.1345	0.091*	
C2	0.1090 (2)	0.3196 (5)	−0.15818 (14)	0.0827 (9)	
H2	0.1062	0.2995	−0.2012	0.099*	
C3	0.1146 (2)	0.5101 (5)	−0.13485 (14)	0.0775 (9)	
H3	0.1165	0.6174	−0.1620	0.093*	
C4	0.11723 (19)	0.5398 (4)	−0.07203 (13)	0.0682 (8)	
H4	0.1203	0.6682	−0.0564	0.082*	
C5	0.11538 (17)	0.3810 (4)	−0.03071 (11)	0.0509 (6)	
C6	0.11088 (18)	0.1917 (4)	−0.05564 (12)	0.0557 (7)	
C7	0.11337 (19)	0.0428 (4)	0.04771 (12)	0.0603 (7)	
C8	0.11338 (17)	0.2423 (3)	0.07410 (11)	0.0497 (6)	
C9	0.11632 (16)	0.3995 (4)	0.03580 (11)	0.0524 (7)	
H9	0.1191	0.5255	0.0533	0.063*	
C10	0.10603 (18)	0.2654 (4)	0.14224 (12)	0.0531 (7)	
C11	0.13845 (18)	0.5046 (4)	0.22323 (11)	0.0535 (7)	
C12	0.16625 (19)	0.3932 (4)	0.27383 (12)	0.0639 (8)	
H12	0.1845	0.2623	0.2686	0.077*	
C13	0.16691 (19)	0.4769 (4)	0.33248 (12)	0.0650 (8)	
H13	0.1863	0.4028	0.3671	0.078*	
C14	0.13875 (19)	0.6712 (4)	0.34023 (11)	0.0567 (7)	
C15	0.11116 (19)	0.7799 (4)	0.28884 (11)	0.0619 (7)	
H15	0.0923	0.9104	0.2938	0.074*	
C16	0.11120 (19)	0.6972 (4)	0.22990 (11)	0.0591 (7)	
H16	0.0929	0.7715	0.1951	0.071*	
C17	0.1323 (2)	0.9462 (4)	0.41084 (12)	0.0645 (7)	
H17A	0.0739	0.9916	0.3926	0.077*	
H17B	0.1819	1.0168	0.3909	0.077*	
C18	0.1356 (2)	0.9869 (4)	0.47999 (11)	0.0675 (8)	
H18A	0.1914	0.9287	0.4984	0.081*	
H18B	0.0831	0.9239	0.4989	0.081*	
C19	0.1343 (2)	1.2047 (4)	0.49476 (12)	0.0707 (8)	
H19A	0.0812	1.2634	0.4731	0.085*	
H19B	0.1893	1.2646	0.4780	0.085*	
C20	0.1304 (2)	1.2574 (4)	0.56327 (12)	0.0723 (8)	
H20A	0.0761	1.1960	0.5806	0.087*	
H20B	0.1843	1.2027	0.5850	0.087*	
C21	0.1269 (2)	1.4752 (5)	0.57543 (13)	0.0831 (9)	
H21A	0.0717	1.5278	0.5546	0.100*	
H21B	0.1797	1.5360	0.5562	0.100*	
C22	0.1265 (2)	1.5383 (5)	0.64278 (13)	0.0839 (9)	
H22A	0.1818	1.4883	0.6641	0.101*	
H22B	0.0734	1.4801	0.6625	0.101*	
C23	0.1229 (3)	1.7629 (5)	0.65022 (15)	0.1031 (12)	
H23A	0.1746	1.8199	0.6287	0.124*	
H23B	0.0666	1.8108	0.6295	0.124*	
C24	0.1255 (3)	1.8364 (6)	0.71544 (16)	0.1332 (16)	
H24A	0.0720	1.7894	0.7365	0.200*	
H24B	0.1256	1.9785	0.7153	0.200*	
H24C	0.1804	1.7889	0.7368	0.200*	

### Atomic displacement parameters (Å^2^)

**Table:**

	*U*^11^	*U*^22^	*U*^33^	*U*^12^	*U*^13^	*U*^23^
O1	0.1120 (16)	0.0356 (11)	0.0641 (12)	−0.0062 (10)	0.0039 (10)	−0.0070 (9)
O2	0.1353 (19)	0.0309 (12)	0.0816 (14)	−0.0007 (11)	0.0057 (12)	0.0064 (10)
O3	0.1055 (16)	0.0459 (12)	0.0723 (12)	−0.0202 (11)	0.0096 (11)	0.0020 (10)
O4	0.0867 (14)	0.0398 (11)	0.0564 (11)	−0.0166 (9)	0.0003 (9)	−0.0004 (9)
O5	0.1129 (17)	0.0487 (13)	0.0570 (12)	0.0036 (10)	−0.0023 (10)	−0.0017 (9)
C1	0.103 (2)	0.059 (2)	0.0653 (19)	−0.0103 (16)	0.0005 (16)	−0.0075 (16)
C2	0.098 (3)	0.090 (3)	0.0596 (18)	−0.0053 (19)	−0.0046 (16)	0.0027 (19)
C3	0.097 (3)	0.066 (2)	0.069 (2)	0.0052 (17)	0.0010 (16)	0.0184 (17)
C4	0.082 (2)	0.0507 (18)	0.0718 (19)	0.0039 (15)	0.0006 (15)	0.0067 (16)
C5	0.0562 (17)	0.0401 (16)	0.0562 (15)	0.0004 (12)	−0.0026 (12)	0.0034 (13)
C6	0.0659 (19)	0.0445 (17)	0.0566 (16)	−0.0040 (13)	−0.0021 (13)	−0.0030 (14)
C7	0.075 (2)	0.0386 (17)	0.0672 (18)	−0.0039 (13)	0.0036 (14)	−0.0036 (15)
C8	0.0573 (17)	0.0307 (14)	0.0608 (16)	−0.0013 (11)	−0.0018 (12)	0.0018 (13)
C9	0.0623 (18)	0.0326 (15)	0.0622 (16)	0.0006 (12)	−0.0020 (12)	−0.0042 (12)
C10	0.0590 (17)	0.0361 (16)	0.0639 (17)	−0.0016 (13)	−0.0025 (13)	0.0025 (14)
C11	0.0697 (19)	0.0365 (15)	0.0542 (15)	−0.0078 (12)	0.0018 (12)	0.0008 (13)
C12	0.087 (2)	0.0373 (15)	0.0670 (18)	0.0048 (14)	−0.0062 (15)	0.0005 (14)
C13	0.092 (2)	0.0435 (17)	0.0588 (17)	0.0071 (14)	−0.0091 (14)	0.0103 (14)
C14	0.0749 (19)	0.0434 (17)	0.0515 (16)	−0.0048 (13)	−0.0026 (13)	0.0046 (13)
C15	0.088 (2)	0.0377 (15)	0.0599 (17)	0.0002 (14)	−0.0029 (14)	0.0023 (13)
C16	0.081 (2)	0.0369 (16)	0.0588 (17)	−0.0038 (13)	−0.0064 (14)	0.0094 (13)
C17	0.077 (2)	0.0548 (19)	0.0615 (17)	0.0054 (14)	0.0026 (13)	−0.0055 (14)
C18	0.069 (2)	0.070 (2)	0.0638 (17)	0.0044 (15)	0.0027 (13)	−0.0025 (15)
C19	0.090 (2)	0.0596 (19)	0.0627 (17)	−0.0049 (16)	0.0010 (15)	−0.0041 (15)
C20	0.086 (2)	0.060 (2)	0.0706 (19)	−0.0015 (15)	0.0053 (15)	−0.0064 (15)
C21	0.106 (3)	0.067 (2)	0.076 (2)	0.0033 (18)	−0.0015 (17)	−0.0039 (17)
C22	0.102 (3)	0.074 (2)	0.077 (2)	0.0031 (18)	0.0093 (17)	−0.0084 (18)
C23	0.152 (3)	0.077 (3)	0.080 (2)	0.015 (2)	0.004 (2)	−0.0115 (19)
C24	0.204 (5)	0.110 (3)	0.088 (3)	0.014 (3)	0.028 (3)	−0.014 (2)

### Geometric parameters (Å, º)

**Table:**

O1—C6	1.376 (3)	C14—C15	1.371 (3)
O1—C7	1.381 (3)	C15—C16	1.379 (3)
O2—C7	1.194 (3)	C15—H15	0.9300
O3—C10	1.198 (3)	C16—H16	0.9300
O4—C10	1.347 (3)	C17—C18	1.503 (3)
O4—C11	1.414 (3)	C17—H17A	0.9700
O5—C14	1.378 (3)	C17—H17B	0.9700
O5—C17	1.422 (3)	C18—C19	1.505 (4)
C1—C6	1.363 (3)	C18—H18A	0.9700
C1—C2	1.374 (4)	C18—H18B	0.9700
C1—H1	0.9300	C19—C20	1.510 (3)
C2—C3	1.381 (4)	C19—H19A	0.9700
C2—H2	0.9300	C19—H19B	0.9700
C3—C4	1.357 (3)	C20—C21	1.496 (4)
C3—H3	0.9300	C20—H20A	0.9700
C4—C5	1.390 (3)	C20—H20B	0.9700
C4—H4	0.9300	C21—C22	1.502 (4)
C5—C6	1.386 (3)	C21—H21A	0.9700
C5—C9	1.427 (3)	C21—H21B	0.9700
C7—C8	1.461 (3)	C22—C23	1.527 (4)
C8—C9	1.342 (3)	C22—H22A	0.9700
C8—C10	1.472 (3)	C22—H22B	0.9700
C9—H9	0.9300	C23—C24	1.479 (4)
C11—C16	1.368 (4)	C23—H23A	0.9700
C11—C12	1.369 (3)	C23—H23B	0.9700
C12—C13	1.375 (3)	C24—H24A	0.9600
C12—H12	0.9300	C24—H24B	0.9600
C13—C14	1.386 (4)	C24—H24C	0.9600
C13—H13	0.9300		
			
C6—O1—C7	123.4 (2)	C15—C16—H16	120.4
C10—O4—C11	121.06 (19)	O5—C17—C18	109.0 (2)
C14—O5—C17	117.82 (19)	O5—C17—H17A	109.9
C6—C1—C2	118.8 (3)	C18—C17—H17A	109.9
C6—C1—H1	120.6	O5—C17—H17B	109.9
C2—C1—H1	120.6	C18—C17—H17B	109.9
C1—C2—C3	120.8 (3)	H17A—C17—H17B	108.3
C1—C2—H2	119.6	C17—C18—C19	112.6 (2)
C3—C2—H2	119.6	C17—C18—H18A	109.1
C4—C3—C2	119.6 (3)	C19—C18—H18A	109.1
C4—C3—H3	120.2	C17—C18—H18B	109.1
C2—C3—H3	120.2	C19—C18—H18B	109.1
C3—C4—C5	120.9 (3)	H18A—C18—H18B	107.8
C3—C4—H4	119.5	C18—C19—C20	115.8 (2)
C5—C4—H4	119.5	C18—C19—H19A	108.3
C6—C5—C4	117.9 (2)	C20—C19—H19A	108.3
C6—C5—C9	117.6 (2)	C18—C19—H19B	108.3
C4—C5—C9	124.5 (2)	C20—C19—H19B	108.3
C1—C6—O1	118.0 (2)	H19A—C19—H19B	107.4
C1—C6—C5	121.8 (2)	C21—C20—C19	113.8 (2)
O1—C6—C5	120.2 (2)	C21—C20—H20A	108.8
O2—C7—O1	116.1 (2)	C19—C20—H20A	108.8
O2—C7—C8	127.4 (2)	C21—C20—H20B	108.8
O1—C7—C8	116.5 (2)	C19—C20—H20B	108.8
C9—C8—C7	119.6 (2)	H20A—C20—H20B	107.7
C9—C8—C10	121.6 (2)	C20—C21—C22	116.5 (3)
C7—C8—C10	118.7 (2)	C20—C21—H21A	108.2
C8—C9—C5	122.6 (2)	C22—C21—H21A	108.2
C8—C9—H9	118.7	C20—C21—H21B	108.2
C5—C9—H9	118.7	C22—C21—H21B	108.2
O3—C10—O4	123.6 (2)	H21A—C21—H21B	107.3
O3—C10—C8	126.3 (2)	C21—C22—C23	112.5 (3)
O4—C10—C8	110.0 (2)	C21—C22—H22A	109.1
C16—C11—C12	121.3 (2)	C23—C22—H22A	109.1
C16—C11—O4	115.6 (2)	C21—C22—H22B	109.1
C12—C11—O4	122.8 (2)	C23—C22—H22B	109.1
C11—C12—C13	119.3 (2)	H22A—C22—H22B	107.8
C11—C12—H12	120.4	C24—C23—C22	115.6 (3)
C13—C12—H12	120.4	C24—C23—H23A	108.4
C12—C13—C14	120.3 (2)	C22—C23—H23A	108.4
C12—C13—H13	119.9	C24—C23—H23B	108.4
C14—C13—H13	119.9	C22—C23—H23B	108.4
C15—C14—O5	125.3 (2)	H23A—C23—H23B	107.4
C15—C14—C13	119.4 (2)	C23—C24—H24A	109.5
O5—C14—C13	115.2 (2)	C23—C24—H24B	109.5
C14—C15—C16	120.5 (3)	H24A—C24—H24B	109.5
C14—C15—H15	119.7	C23—C24—H24C	109.5
C16—C15—H15	119.7	H24A—C24—H24C	109.5
C11—C16—C15	119.2 (2)	H24B—C24—H24C	109.5
C11—C16—H16	120.4		
			
C6—C1—C2—C3	0.6 (5)	C9—C8—C10—O3	148.8 (3)
C1—C2—C3—C4	−1.0 (5)	C7—C8—C10—O3	−28.5 (4)
C2—C3—C4—C5	0.7 (4)	C9—C8—C10—O4	−28.8 (3)
C3—C4—C5—C6	0.1 (4)	C7—C8—C10—O4	153.9 (2)
C3—C4—C5—C9	−178.9 (2)	C10—O4—C11—C16	−131.1 (3)
C2—C1—C6—O1	−179.0 (3)	C10—O4—C11—C12	55.5 (3)
C2—C1—C6—C5	0.2 (4)	C16—C11—C12—C13	−0.1 (4)
C7—O1—C6—C1	−179.5 (2)	O4—C11—C12—C13	172.9 (2)
C7—O1—C6—C5	1.2 (4)	C11—C12—C13—C14	0.7 (4)
C4—C5—C6—C1	−0.6 (4)	C17—O5—C14—C15	−11.9 (4)
C9—C5—C6—C1	178.5 (2)	C17—O5—C14—C13	167.5 (2)
C4—C5—C6—O1	178.7 (2)	C12—C13—C14—C15	−0.7 (4)
C9—C5—C6—O1	−2.3 (4)	C12—C13—C14—O5	179.8 (2)
C6—O1—C7—O2	−176.5 (2)	O5—C14—C15—C16	179.5 (2)
C6—O1—C7—C8	1.8 (4)	C13—C14—C15—C16	0.1 (4)
O2—C7—C8—C9	174.3 (3)	C12—C11—C16—C15	−0.5 (4)
O1—C7—C8—C9	−3.8 (4)	O4—C11—C16—C15	−174.0 (2)
O2—C7—C8—C10	−8.4 (4)	C14—C15—C16—C11	0.5 (4)
O1—C7—C8—C10	173.6 (2)	C14—O5—C17—C18	178.6 (2)
C7—C8—C9—C5	2.8 (4)	O5—C17—C18—C19	174.9 (2)
C10—C8—C9—C5	−174.5 (2)	C17—C18—C19—C20	175.7 (2)
C6—C5—C9—C8	0.3 (4)	C18—C19—C20—C21	−178.6 (3)
C4—C5—C9—C8	179.2 (3)	C19—C20—C21—C22	−177.7 (3)
C11—O4—C10—O3	−4.6 (4)	C20—C21—C22—C23	180.0 (3)
C11—O4—C10—C8	173.1 (2)	C21—C22—C23—C24	−178.1 (3)

### Hydrogen-bond geometry (Å, º)

**Table:**

*D*—H···*A*	*D*—H	H···*A*	*D*···*A*	*D*—H···*A*
C4—H4···O1^i^	0.93	2.59	3.513 (4)	174
C9—H9···O2^i^	0.93	2.51	3.420 (3)	167
C16—H16···O2^ii^	0.93	2.71	3.551 (3)	151
C16—H16···O3^ii^	0.93	2.63	3.338 (3)	133

Symmetry codes: (i) *x*, *y*+1, *z*; (ii) −*x*, −*y*+1, −*z*.
